# Identification of Differentially Expressed Genes Associated with Litter Size in Berkshire Pig Placenta

**DOI:** 10.1371/journal.pone.0153311

**Published:** 2016-04-14

**Authors:** Seul Gi Kwon, Jung Hye Hwang, Da Hye Park, Tae Wan Kim, Deok Gyeong Kang, Kyung Hee Kang, Il-Suk Kim, Hwa Chun Park, Chong-Sam Na, Jeongim Ha, Chul Wook Kim

**Affiliations:** 1 Swine Science and Technology Center, Gyeongnam National University of Science & Technology, Jinju, South Korea; 2 Department of Animal Resource Technology, Gyeongnam National University of Science & Technology, Jinju, South Korea; 3 Dasan Pig Breeding Co., Namwon, South Korea; 4 Department of Animal Biotechnology, Chonbuk National University, Jeonju, South Korea; University of Connecticut, UNITED STATES

## Abstract

Improvement in litter size has become of great interest in the pig industry because fecundity is directly related to sow reproductive life. Improved reproduction has thus been achieved by elucidating the molecular functions of genes associated with fecundity. In the present study, we identified differentially expressed genes (DEGs) via transcriptomic analysis using RNA-sequencing (RNA-Seq) in Berkshire pig placentas from larger (LLG, mean litter size >12) and smaller (SLG, mean litter size < 6.5) litter size groups. In total 588 DEGs were identified (*p* < 0.05, > 1.5-fold change), of which 98 were upregulated, while 490 were downregulated in the LLG compared with the SLG. Gene Ontology (GO) enrichment was also performed. We concluded that 129 of the 588 DEGs were closely related to litter size according to reproduction related genes selected based on previous reports, as 110 genes were downregulated and 19 upregulated in the LLG compared with the SLG. RT-qPCR utilizing specific primers targeting the early growth response 2 (*EGR2*), pheromaxein c subunit (*PHEROC*) and endothelial lipase (*LIPG*) genes showed high accordance with RNA-Seq results. Furthermore, we investigated the upstream regulators of these three genes in the placenta. We found that *WNT9B*, a Wnt signaling pathway molecule, and *IL-6*, known inducers of *EGR2* and *LIPG*, respectively, were significantly increased in LLG compared with SLG. We believe that the induction of *IL-6* and *LIPG* may play an important role in increasing nutrition supply through the placenta from the sow to the piglet during gestation. These results provide novel molecular insights into pig reproduction.

## Introduction

Improved litter size is a principal interest in the pig industry and among breeders. Litter size is a complex trait composed of many subordinated traits, such as ovulation rate, embryonic/fetal survival, uterine capacity, and others. Moreover, since selection by litter size has its limitations, including low heritability and sex-restriction, various efforts have been made to identify factors influencing litter size. These efforts have included optimizing nutrition and husbandry, management of sows, and genetic factors [1]. Genetic selection for increased litter size has in turn increased the prolificacy of sows and has drastically improved over the past 10 years, resulting in an average increase of 1.8 piglets per litter [2].

The first successful evidence of a candidate gene associated with litter size was estrogen receptor 1 (*ESR1*), which was identified using restriction fragment length polymorphism (RFLP) analysis [3]. Retinol-binding protein 4 (*RBP4*), erythropoietin receptor (*EPOR*), amphiregulin (*AREG*), epidermal growth factor (*EGF*), and others have also been determined to be associated with litter size [1, 4–6]. In addition, several expression studies performed in pigs have developed a profile of differentially expressed genes (DEGs) and even microRNAs (miRNAs) in endometria, ovaries and placentas [7–9]. The mRNA of the oligoadenylate synthetase 1 (*OAS1*) gene was highly expressed, whereas that of the *RBP4*, thiosulfate sulfurtransferase (*TST*) and vitronectin (*VTN*) genes were lowly expressed in the ovaries of a high prolificacy group compared with a low prolificacy group [7]. Moreover, the investigation uncovered the location of candidate genes according to significant quantitative trait loci (QTL) previously reported by Noguera et al. [7, 10]. The porcine endometrial transcriptome was also analyzed during the implantation period [8]. The androgen receptor (AR) was revealed to have a close interaction with genes associated with important processes during early pregnancy [8]. Recent reports on differential expression of miRNAs determined that selected miRNAs affect cell growth, angiogenesis and trophoblast differentiation, and these impacts may be important for placental growth and function [9]. Moreover, fetal loss in the pig is mostly a result of decreased uterine capacity and placental efficiency [11, 12]. Therefore, to understand the molecular mechanisms by which the structure and function of the placenta may be regulated is essential to increasing litter size [11]. Historically, the Chinese Meishan pig breed is known for its prolificacy and large litter size [13]. Some evidence also suggests that the Chinese Meishan may have improved uterine capacity and placental efficiency [14]. However, the reproductive rate of Berkshire pigs is lower than that of other pig breeds, although the production of Berkshire pigs has been increasing recently [15]. Improvement in the litter size of Berkshire pigs is essential for Berkshire pig breeders. In this context, we hypothesized that DEGs in the placenta play a critical role in the determination of litter size. However, the placental transcriptome of Berkshire pigs has not yet been elucidated. Therefore, this study analyzed the RNA-Seq of Berkshire placentas, which revealed novel genes that may be involved in placental development and function, thus playing a role in determining litter size. These results provide molecular insights into the genes underlying pig fecundity.

## Materials and Methods

### Ethics statement

There is an exception to the application of the Enforcement Rule of the Korean Animal Protection Act from Article 23 in the case of experiments conducted for scientific purposes concerning the relevant animal, ecology, and habitude of species. We did not need to obtain approval from the Animal Care and Use Committee of Gyeongnam National University of Science and Technology (ACUC of GNTECH) because all of the placentas used in this study were a natural by-product. Moreover, no ethics committee approval for the study was required for livestock in the Republic of Korea. We have enclosed the ACUC of GNTECH waiver (Supplementary information). The pigs used in this study were raised according to the guidelines on animal care and use established by ACUC of GNTECH and with the Korean Animal Protection Act and related laws.

### Placenta collection

Berkshire placentas were collected by a trained veterinarian from sows reared in the same environment (Dasan Pig Breeding Company, South Korea). We divided the placentas into two groups according to litter size: the larger (LLG) and smaller (SLG) litter size groups. The LLG was defined by an estimated breeding value (EBV) ≥ 0.8 and litter size ≥ 12, whereas the SLG was selected by an EBV ≤ –0.7 and litter size ≤ 6.5. Detailed information for sows is described in Table 1. Farrowing was allowed to start naturally. After delivery, the placental tissues were collected as part of the routine care of pigs. Samples were excised from the maternal side of the placenta 2 cm from the umbilical cord insertion site and free of maternal decidua corresponding to the chorioallantoic placenta. After collection, placental tissues were rinsed with PBS and frozen rapidly in liquid nitrogen.

**Table 1:** Characteristics of the Berkshire pigs used in this study.

Group	sample ID	months of age	body weight	total parity numbers	mean of litter size	EBV
SLG	SLG1	23	175	4	5.5	-0.78
	SLG2	23	170	4	6	-0.8
	SLG3	25	180	4	6.5	-0.81
LLG	LLG1	27	180	4	12.25	0.9
	LLG2	28	200	4	12	0.8
	LLG3	28	200	3	12	0.85

### RNA isolation and RNA-Seq

Total RNA was isolated from three Berkshire pig placentas in each group using TRIzol reagent according to the manufacturer’s instructions (Molecular Research Center, Cincinnati, OH, USA). We pooled the isolated RNA from the three different pigs in a ratio of 1:1:1 before library preparation. The quality of the resulting total RNA was measured using an Agilent 2100 Bioanalyzer (Agilent Technologies, Santa Clara, CA, USA). All extractions exhibited an RNA integrity number > 7.0 and a 28S:18S ratio > 1.0.

RNA-Seq libraries were prepared using a TruSeq RNA Sample Prep kit (Illumina, San Diego, CA, USA). The poly A-containing mRNA molecules were purified from 2 μg total RNA from each sample using poly-T oligo-attached magnetic beads. The mRNA was fragmented into approximately 200 base pair pieces. Using reverse transcriptase and random hexamer primers, the first-strand cDNA was synthesized from the mRNA fragments. Second-strand cDNA was then synthesized using DNA polymerase I and RNaseH. The end of the cDNA fragment was then subjected to an end repair process that included the addition of a single ‘A’ base, followed by ligation of the adapters. Products were purified and enriched by polymerase chain reaction (PCR) to amplify the library DNA. Libraries were quantified using a KAPA Library Quantification kit (KAPA Biosystems, South Africa) and an Agilent 2100 Bioanalyzer. After quantitative reverse transcription-polymerase chain reaction (RT-qPCR) validation, libraries were subjected to paired-end sequencing with 100-base pair read lengths using the Illumina HiSeq 2500 platform (Illumina).

### RNA-Seq analysis

High-quality reads were obtained by removing adaptor sequences, reads in which the percentage of unknown bases was greater than 10% or low quality reads (more than 20% of <Q20 bases). Selected reads were aligned to the susScr3 reference sequence using STAR (version 2.3.0e) [16], and the gene description in the Ensembl gene database (version 72) [17] was used to increase the accuracy of quantification. Expression levels were quantified using htseq-count (ver. 0.5.4p3) [18], while considering intersection-nonempty rules and paired-end alignments. DEGs were analyzed using TCC [19] with the negative binomial (NB) statistical test available in two R packages iDEGES/edgeR and cutoff values of *p* < 0.05 and 1.5-fold change.

### Pearson correlation and functional enrichment analysis

A Pearson correlation coefficient was computed for fragments per kilobase of exon per million fragments mapped (FPKM) and log_2_ (FPKM) values for genes from each group. Gene ontology (GO) [17] annotation and KEGG pathway analysis were performed on all DEGs using the Database for Annotation, Visualization and Integrated Discovery tool (DAVID; National Institute of Health; http://david.abcc.ncifcrf.gov/) Bioinformatics Resources 6.7. Statistics were applied for the selection of GO categories using a modified Fisher’s exact *p*-value < 0.01 (EASE score in DAVID).

### RT-qPCR

Total RNA was isolated from three different placentas and was used for RT-qPCR. RNA (1 μg) was then used for reverse transcription in a 20-μL reaction volume using Superscript II (Invitrogen). RT-qPCR was performed using a Rotor Gene Q Thermocycler (QIAGEN) with SYBR green. Gene-specific exon spanning primers were designed using the Primer3 program as described previously (S1 Table) [20]. RT-qPCR was performed in a 10-μL reaction volume containing 1 μL cDNA (50 ng) as the template, 5 μL Rotor Gene SYBR Green PCR Master Mix, 1 μL each of the forward and reverse primers (10 pmol), and 3 μL H_2_O using the following cycling parameters: 40 cycles at 94°C for 5 s and 60°C for 10 s. A melting curve was performed at the end of the PCR for 5 s from 60°C to 95°C to identify unique PCR products amplified during the reaction. A no template control was used as a negative control. The qPCR reaction was repeated three times independently. *PPIA* was used as a reference gene [21]. Amplification efficiencies and correlation coefficients (R^2^ values) were generated using the slopes of standard curves obtained from serial dilutions. Standard curves with a 10-fold dilution series were used to calculate the amplification efficiency. The amplification efficiency was calculated according to the formula: efficiency (%) = (10^(-1/slope)^-1) × 100. The efficiency of all of the RT-qPCR amplifications was almost 90%. Data were analyzed by the relative quantification method using 2^-ΔΔCt^. The control sample used to determine fold induction was the lowest expressed sample for the target gene. The significance of differences was analyzed using Student’s t-test or the Mann–Whitney test with *p* < 0.05 considered significant.

## Results

### RNA-Seq analysis

**Fig 1:**
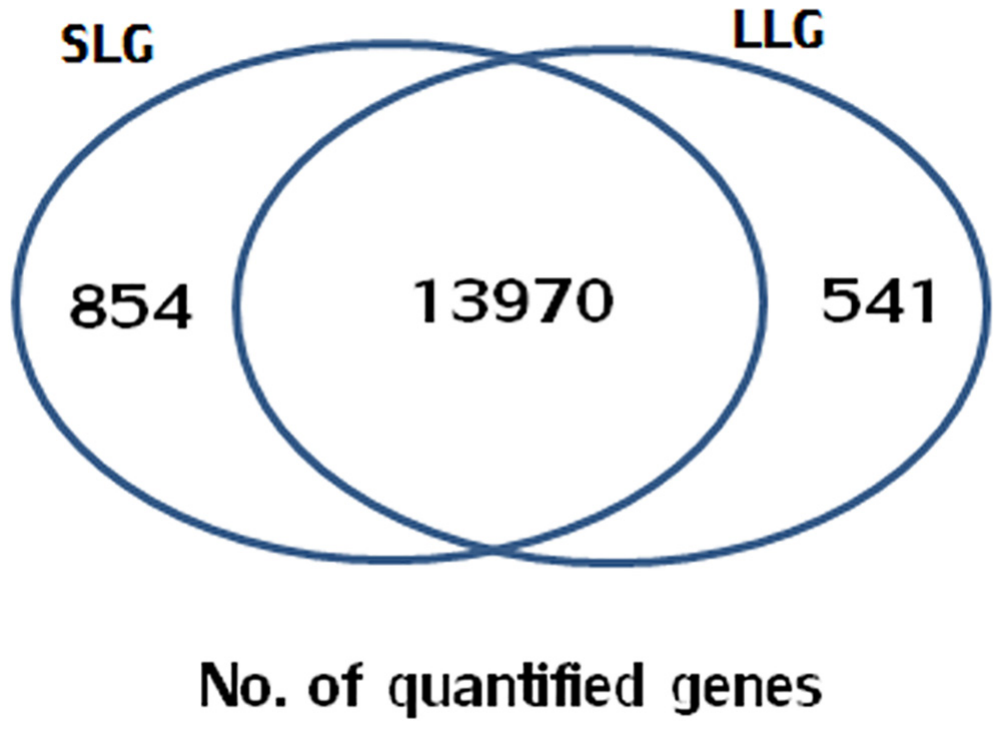
Identification of DEGs by RNA-Seq analysis. The number of annotated genes using RNA-seq is shown. A total of 14,511 genes in the LLG and 14,824 genes in the SLG were detected. The number of commonly expressed genes among both groups was 13,970.

In this study, DEGs were identified from Berkshire pig placentae, and the role of candidate genes influencing litter size was investigated. To identify DEGs, we divided the placentae into two groups (LLG and SLG) according to litter size. RNA-Seq was performed on total RNA pooled from three placentae of Berkshire pigs. The total numbers of clean reads were 47,289,182 (90.8%) and 45,580,292 (95.3%) and mapped reads were 40,421,582 (85.5%) and 39,219,270 (86.0%) in LLG and SLG, respectively (data not shown). The number of genes expressed was quantified using htseq-count and was 14,824 in the LLG and 14,511 in the SLG. The number of genes commonly expressed between the two groups was 13,970 (Fig 1).

### Analysis of annotated genes

Pearson correlation coefficients were calculated for each group. The Pearson correlation coefficient between the FPKM of genes from each group was 0.962. Similarly, the Pearson correlation coefficient between the log_2_(FPKM) of genes was 0.978. These results indicate a strong correlation between the two groups.

### Analysis of DEG and GO enrichment analysis

**Fig 2:**
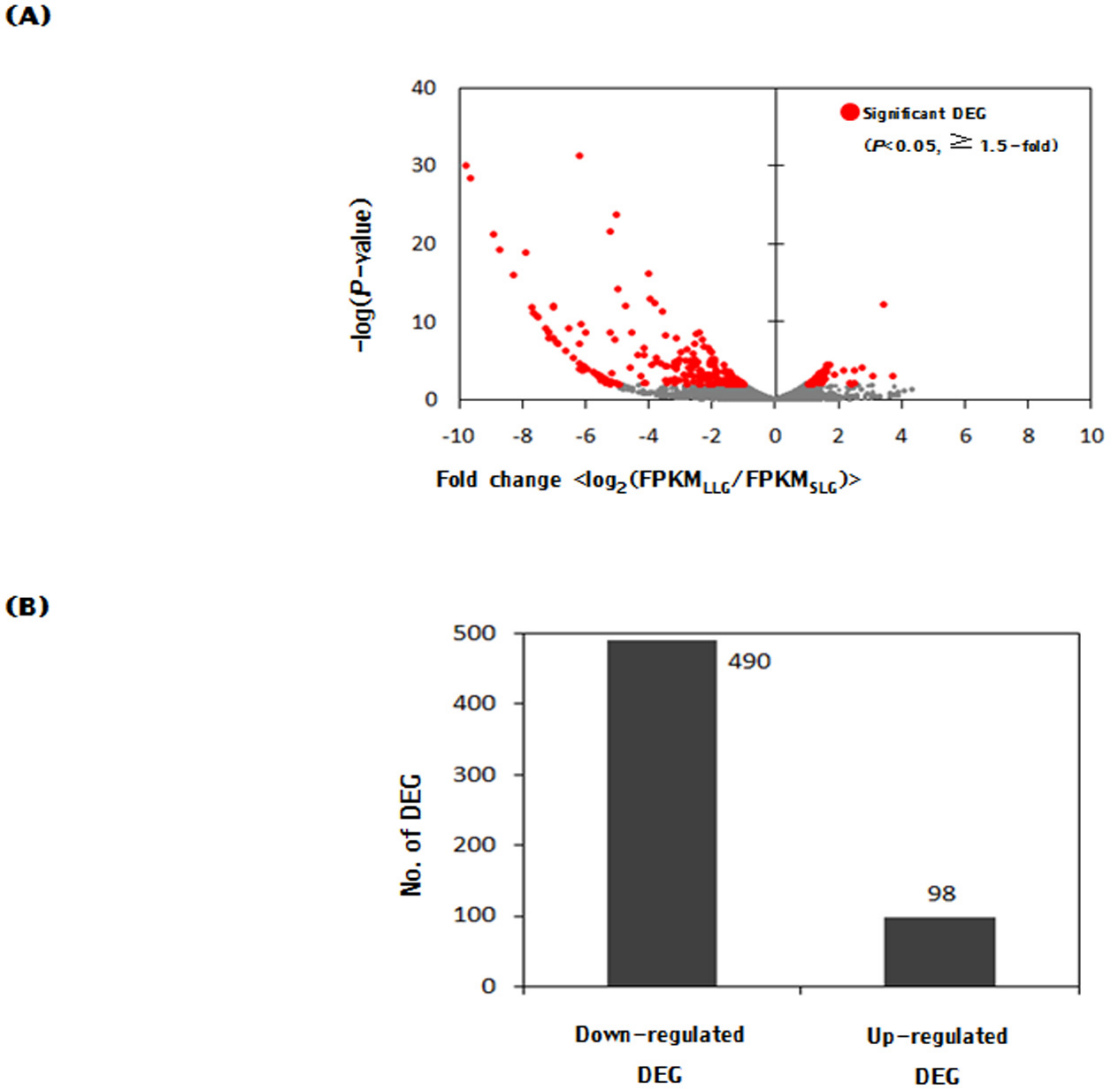
Distribution of DEGs. (A) The dot graph shows each DEG between the LLG and SLG. log_2_-fold change between fragments per kilobase of exon per million reads mapped (FPKM) in the SLG and LLG according to the Cufflinks package are presented along the *x* axis. Red dots represent significantly altered DEGs between the LLG and SLG (*p* < 0.05, fold change > 1.5, the decimal system). The *y* axis indicates significance as -log (*p* value). (B) The number of downregulated and upregulated DEGs in the LLG compared with the SLG.

**Fig 3:**
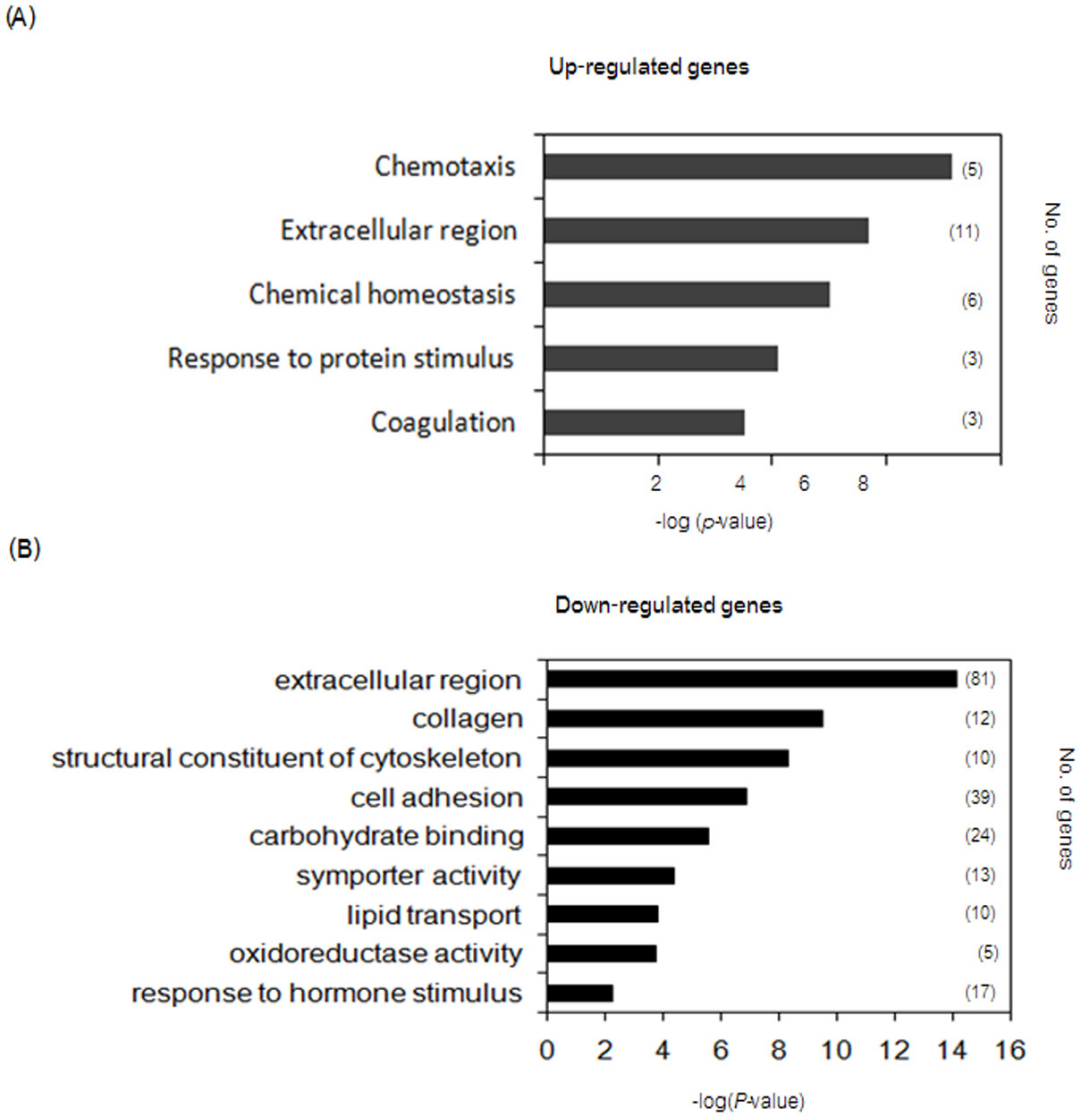
GO enrichment analysis. GO enrichment analysis was performed for the upregulated (A) and downregulated (B) DEGs. The *x* axis indicates the significance of DEGs as −log (*p* value). The number of genes in each category is shown on the *y* axis, as indicated in parentheses.

Each gene annotated as a DEG is shown as a dot graph in Fig 2A. The number of downregulated DEGs was higher than the number of upregulated DEGs in the LLG compared with the SLG. The significantly altered DEGs are indicated as red dots. A total of 490 and 98 genes were annotated among the downregulated DEGs and upregulated DEGs in the LLG, respectively (Fig 2B). Next, GO enrichment analysis was performed on the upregulated genes in the LLG. The annotated genes were associated with chemotaxis, extracellular region, chemical homeostasis, response to protein stimulus and coagulation. Eleven genes were associated with the extracellular region, including *PLAT*, *INHBA*, *CCL2* and *IL8*. Six genes were associated with chemical homeostasis, including *ALAS2*, *CCL2* and *EGR2*. Five genes were associated with chemotaxis, including *PLAU* and *PLAUR* (Fig 3A). We next compared GO enrichment analysis results with the downregulated genes in the LLG. The genes were categorized into extracellular region, collagen, structural constituent of cytoskeleton, cell adhesion, carbohydrate binding, symporter activity, lipid transport, oxidoreductase activity and response to hormone stimulus. Eighty-one genes were associated with the extracellular region, including *LTBP2* and *FAM3D*. In addition, 39 genes were associated with cell adhesion, while 24 genes were related to carbohydrate binding (Fig 3B).

### Analysis of DEGs associated with litter size

Next, we investigated the genes associated with litter size among all DEGs by searching for terms related to litter size such as reproduction, fecundity, prolificacy and litter size in the NCBI PubMed. A total of 129 (22%) of the 588 DEGs were selected. The upregulated DEGs included 19 genes; whereas, the downregulated DEGs included 110 genes (S2 and S3 Tables). The representative genes are listed in Table 2. Among the upregulated DEGs, *PHEROC* and *XIRPL* levels showed approximately 5.3- to 5.6-fold change in the LLG compared with the SLG, with the decimal system (Table 2). Interestingly, *KRT1* was decreased more than 1,200-fold in the LLG compared with the SLG (S3 Table). Of note, some genes described as downregulated DEGs, such as *USH1C*, *LCN15*, *DAO*, *MUC13A*, *REG4*, *EPS8L3*, *KRT77*, *SLC6A19*, *APO8*, *HNF4A*, *LGALS2*, *IGSF23*, *RBP2* and *TM4SF20*, were determined to lack transcripts in the LLG. Presumably, these genes might play a negative role in reproduction regulation.

**Table 2:** List of DEGs between LLG and SLG.

Accession	Symbol	Description	log_2_fc	*p*-value
ENSSSCG00000013067	*PHEROC*	pheromaxein C subunit	2.51	0.01
ENSSSCG00000011265	*XIRP1*	xin actin-binding repeat containing 1	2.42	0.01
ENSSSCG00000011951	*NFKBIZ*	nuclear factor of kappa light polypeptide	1.85	< .001
ENSSSCG00000010224	*EGR2*	early growth response 2	1.62	< .001
ENSSSCG00000003451	*PDPN*	Podoplanin	1.55	< .001
ENSSSCG00000004509	*LIPG*	endothelial lipase	1.51	< .001
ENSSSCG00000011663	*RBP2*	retinol binding protein 2, cellular	Only SLG	< .001
ENSSSCG00000009558	*F10*	coagulation factor X protein	-5.16	< .001
ENSSSCG00000011453	*ITIH4*	inter-alpha-trypsin inhibitor heavy chain H4	-4.99	< .001
ENSSSCG00000020694	*DSG1*	desmoglein 1	-4.36	< .001
ENSSSCG00000016196	*VIL1*	Villin-1	-4.18	< .001
ENSSSCG00000023949	*PIM3*	pim-3 oncogene	-3.50	< .001
ENSSSCG00000007507	*PCK1*	phosphoenolpyruvate carboxykinase 1	-3.47	< .001

Note: log_2_fc means the log_2_ value of the fold change in fragments per kilobase of exon per million fragments mapped (FPKM) by comparing of LLG with SLG.

### Validation of DEGs associated with litter size by RT-qPCR

**Fig 4:**
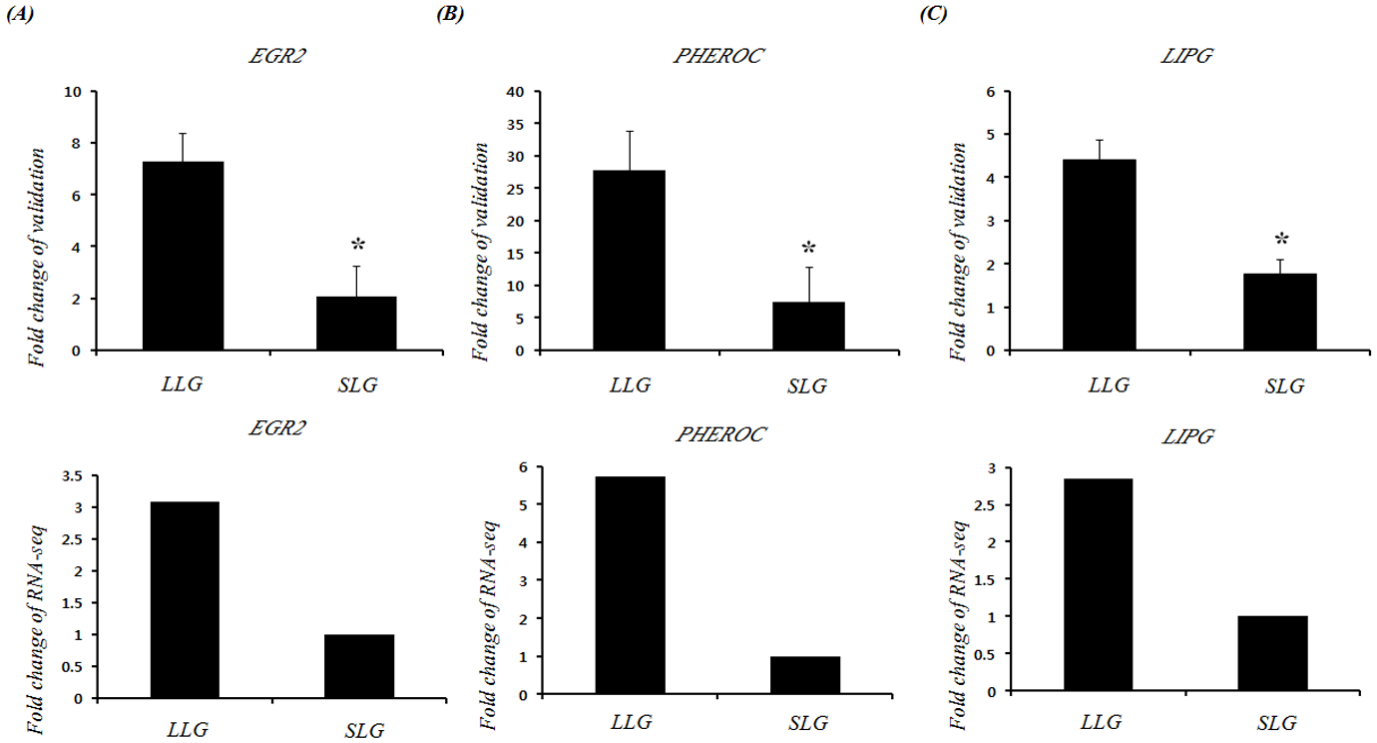
RT-qPCR analysis of *EGR2*, *PHEROC* and *LIPG* in placental tissue. Three different placentas from LLG and SLG were prepared immediately following delivery and subjected to RT-qPCR analysis. The fold change of validation (upper panel) and RNA-Seq (lower panel) for the (A) *EGR2*, (B) *PHEROC* and (C) *LIPG* genes are presented. The RT-qPCR data were analyzed by relative quantification using 2^-ΔΔCt^. Data are expressed as means ± standard deviation (S.D.). Each experiment was performed in triplicate. *, *p* < 0.05 versus SLG.

Next, RT-qPCR was performed to confirm the RNA-Seq results. The results from RT-qPCR were highly accordant with those from RNA-Seq. PCR products amplified using primers specific to early growth response 2 (*EGR2*), pheromaxein c subunit (*PHEROC*) and endothelial lipase (*LIPG*) are shown in Fig 4. In all cases, the relative fold changes in gene expression were in the same direction as those observed in the RNA-Seq results, and consistently showed a higher magnitude with RT-qPCR than RNA-Seq technology (Fig 4).

**Fig 5:**
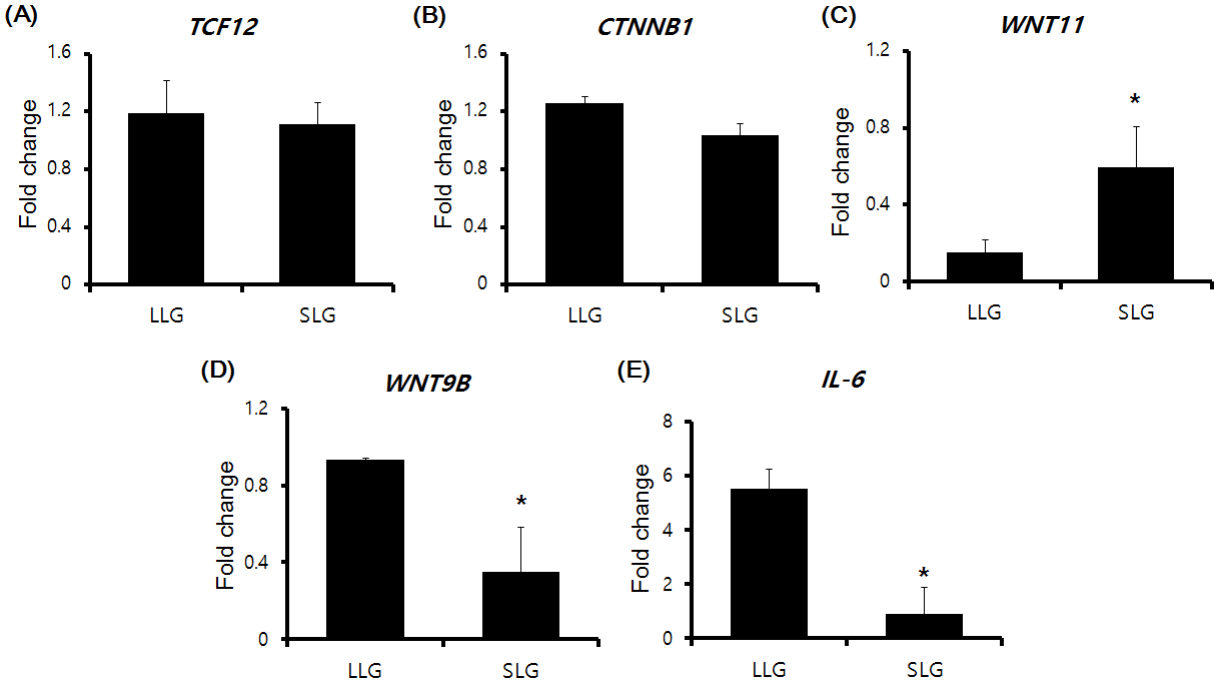
RT-qPCR analysis to assess *TCF12*, *CTNNB1*, *WNT11*, *WNT9B* and *IL-6* mRNA expression. RNAs from three different individual placentas each were prepared from LLG and SLG. RT-qPCR was performed using gene-specific primers. *PPIA* was used as a universal control. The RT-qPCR data were analyzed by relative quantification using 2^-ΔΔCt^. Data are expressed as means ± standard deviation (S.D.). Each experiment was performed in triplicate. *, *p* < 0.05 versus SLG.

To examine the molecules upstream from the DEGs annotated in this study, we investigated the mRNA expression of genes related to the Wnt signaling pathway such as *TCF12*, *CTNNB1*, *WNT11* and *WNT9B*, because Wnt signaling plays a crucial role in placenta and trophoblast development and differentiation [22]. In addition, several Wnt ligands are strongly expressed in first trimester trophoblasts, suggesting that these ligands could play a crucial role in early placental function and development [23]. Moreover, some evidence of *EGR2* induction via the Wnt signaling pathway has been obtained in investigations in other fields [24–26]. When we examined gene expression in three different placental RNA samples from LLG and SLG using RT-qPCR, the mRNA levels of *TCF12* and *CTNNB1* were unchanged between the two groups (Fig 5A and 5B). In comparison, the expression of *WNT11* mRNA was decreased and that of *WNT9B* was increased in LLG compared with SLG, respectively (Fig 5C and 5D).

Nutrient supply is a crucial factor supporting piglet growth. The primary fetal nutrients that cross the placenta are glucose, free amino acids, ketone bodies, and free fatty acids [27]. The triglyceride lipase gene (TLG) family comprises secreted lipases that hydrolyze triglycerides and phospholipids [28]. LIPG is one of the most well studied lipases in the TLG family [28]. Of note, *LIPG* is expressed in trophoblasts in the first trimester in humans. The expression of *LIPG* mRNA was significantly lower in placentas from intrauterine growth restricted pregnancies compared with normal placentas [28]. In this regard, the upregulation of *LIPG* may accelerate nutrient supply via the placenta, strongly suggesting that LIPG contributes to increased litter size. A limited number of cytokines and hormones, such as TNF-α, IL-6, leptin, and adiponectin, have been determined to regulate *LIPG* mRNA expression [28–30]. The mRNA expression of TNF-α, leptin and adiponectin were unchanged between the LLG and SLG (data not shown). However, *IL-6* was significantly increased in the LLG compared with the SLG (Fig 5E). Moreover, one of the pro-inflammatory cytokines, *IL-1B* was also upregulated in LLG compared with SLG (data not shown). IL-6, together with IL-1β might enhance the capacity of endothelial cells to bind and transport high-density lipoproteins, and could increase the permeability of the endothelial barrier to allow easy transport of nutrients across the placenta [31].

## Discussion

Various research groups have investigated DEGs from reproductive tissues such as the endometrium in pigs [32, 33]. DEGs in the placenta have rarely been demonstrated using RNA-Seq. In this study, we revealed DEGs according to litter size in Berkshire pig placentas. We found 588 annotated DEGs between LLG and SLG. A total of 129 genes (19 upregulated genes and 110 downregulated genes in LLG compared with SLG) from the 588 DEGs were selected because they are associated with litter size [34–37]. Intriguingly, in an effort to delineate the molecular pathways responsible for the upregulation of *EGR2* and *LIPG*, we discovered that the induction of *WNT9B* and *IL-6* might stimulate the upregulation of *EGR2* and *LIPG*, respectively, in LLG compared with SLG. Previously, Wnt signaling is known to increase *EGR2* expression [23–25]. Moreover, the activation of Wnt signaling accelerates *EGR2* expression and compromises the inhibitory effect of glucocorticoids on *EGR2* expression [25]. Therefore, we evaluated the mRNA expression of the following Wnt ligands: *WNT6*, *WNT7B WNT9B*, *WNT10A*, and *WNT11*. *WNT9B* was upregulated in the LLG compared with the SLG, while no expression of *WNT6*, *WNT7B* or *WNT10A* mRNA was detected, and *WNT11* was decreased in the LLG compared with the SLG. Although we could not exclude a role of *WNT11* in SLG, *WNT9B* is a likely mediator of *EGR2* induction in LLG. As we mentioned above, Wnt signaling plays a critical role in development and differentiation of the placenta and trophoblast. Wnt may also modulate other trophoblast processes such as phospholipid uptake and transport. However, further investigation is needed to fully understand the role of Wnt signaling in the placenta.

Since the pig industry is actively incorporating marker-associated selection to improve economical traits such as litter size, we searched for SNPs of the DEGs in this study. Four different missense SNPs were identified in the *EGR2* gene. A future study should examine the correlation of polymorphisms in *EGR2* and litter size, although we are not convinced whether the polymorphisms affect the mRNA expression or not.

Together with the RNA-Seq experiment, we originally performed whole genome bisulfite sequencing (WGBS) to analyze the correlation between DNA methylation and mRNA expression. DNA methylation is a process that adds methyl groups to DNA, which stably alters the expression of genes. Some methylation is heritable. Therefore, we believe that DNA markers in which the nucleotide sequence is not changed, but influences gene expression could be useful when breeders select breeding pigs. We also wanted to determine whether the differentially methylated region (DMR) within specific genes of interest could regulate their mRNA expression. For example, *PCK1* was hypomethylated in gene body region (decrease methylated status) in LLG compared with SLG, and the expression of *PCK1* mRNA was down-regulated in LLG compared with SLG, which indicates a correlation between DNA methylation and mRNA expression (in press).

In conclusion, we identified DEGs associated with litter size from Berkshire pig placentas that may play crucial roles in pig prolificacy. The results of this study can provide important molecular insights into regulation of litter size. More detailed investigations will be necessary in the future, including SNP analysis of candidate genes, for selection of high prolificacy pigs in order to apply this knowledge to the pig industry.

## Supporting Information

S1 Table Primer sequences for RT-qPCR used in the study. (DOCX)

S2 Table List of up-regulated DEGs related to fecundity in LLG compared with SLG. (DOCX)

S3 Table List of down-regulated DEGs related to fecundity in LLG compared with SLG. (DOCX)
