# Supplementary material for: Identification of Differentially Expressed Genes Associated with Litter Size in Berkshire Pig Placenta
Source: PLoS One. 2016 Apr 14;11(4):e0153311. doi: 10.1371/journal.pone.0153311 (PMC4831801; doi:10.1371/journal.pone.0153311)
Supplement: S1 Table — (DOCX) [file pone.0153311.s001.docx]

**S1 Table. primer sequences for RT-qPCR used in the study.**

|  | Accession No. | Gene name |  | Primer sequence |  |
| --- | --- | --- | --- | --- | --- |
|  | NM_001097488.1 | *EGR2* |  | F : TCGGTGACCATCTTTCCCAA | |
|  |  |  |  | R : GGGTCAATGGAGAACTTGCC | |
|  | NM_001123161.1 | *PHEROC* |  | F : CCGTTTACTGCTCTGCAGGTTCTG | |
|  |  |  |  | R : TCTTCTGACTGCTTGAGAAAACA | |
|  | NM_001243029.1 | *LIPG* |  | F : TGCAACAGCCAAAACCTTCT | |
|  |  |  |  | R : TGTCCCACTTTCCTCGTGTT | |
|  | XM_005656681.1 | *IL-6* |  | F : GCCTGGAAGAAGATGCCAAA | |
|  |  |  |  | R : GCCAGTACCTCCTTGCTGTT | |
|  | XM_013992945.1 | *TCF12* |  | F : ACTGGGAAGCAGTCAGTTCA | |
|  |  |  |  | R : GGCCCCTAATCGACTGTCA | |
|  | NM_214367.1 | *CTNNB1* |  | F : GATCAAAACTCGCTCCGTGG | |
|  |  |  |  | R : TGCTTAGCTTCAATACAGGCAAT | |
|  | XM_005667138.2 | *WNT11* |  | F : GGGAGTCAGCCTTCGTGTAT | |
|  |  |  |  | R : TGTCTCCCCACTTCACTGTT | |
|  | XM_013980866.1 | *WNT9B* |  | F : CTGGCACCTTCATCCACCTA | |
|  |  |  |  | R : GGAACTGAAACTGGCACTCG | |
|  | NM_214353.1 | *PPIA* |  | F : CACAAACGGTTCCCAGTTTT | |
|  |  |  |  | R : TGTCCACAGTCAGCAATGGT | |
