# Supplementary material for: Identification of Differentially Expressed Genes Associated with Litter Size in Berkshire Pig Placenta
Source: PLoS One. 2016 Apr 14;11(4):e0153311. doi: 10.1371/journal.pone.0153311 (PMC4831801; doi:10.1371/journal.pone.0153311)
Supplement: S2 Table — (DOCX) [file pone.0153311.s002.docx]

**S2 Table. List of up-regulated DEGs related to fecundity in LLG compared with SLG**

| **Accession** | **Symbol** | **Description** | **log_2_fc** | ***p*-value** |
| --- | --- | --- | --- | --- |
|  |  |  |  |  |
| ENSSSCG00000013067 | *PHEROC* | pheromaxein C subunit | 2.51 | 0.01 |
| ENSSSCG00000011265 | *XIRP1* | xin actin-binding repeat containing 1 | 2.42 | 0.01 |
| ENSSSCG00000011951 | *NFKBIZ* | nuclear factor of kappa light polypeptide gene enhancer in B-cells inhibitor, zeta | 1.85 | < .001 |
| ENSSSCG00000008963 | *AREG* | amphiregulin | 1.79 | 0.02 |
| ENSSSCG00000010224 | *EGR2* | early growth response 2 | 1.62 | < .001 |
| ENSSSCG00000003451 | *PDPN* | podoplanin | 1.55 | < .001 |
| ENSSSCG00000004509 | *LIPG* | lipase, endothelial | 1.51 | < .001 |
| ENSSSCG00000011877 | *CD86* | CD86 molecule | 1.48 | < .001 |
| ENSSSCG00000003065 | *PLAUR* | plasminogen activator, urokinase receptor | 1.39 | < .001 |
| ENSSSCG00000009482 | *SPRY2* | sprouty RTK signaling antagonist 2 | 1.37 | < .001 |
| ENSSSCG00000015476 | *CHI3L1* | chitinase 3-like 1 (cartilage glycoprotein-39) | 1.26 | < .001 |
| ENSSSCG00000011208 | *ZNF385D* | zinc finger protein 385D | 1.18 | 0.01 |
| ENSSSCG00000007032 | *PLAT* | plasminogen activator, tissue | 1.18 | < .001 |
| ENSSSCG00000007978 | *HBA* | hemoglobin subunit alpha | 1.14 | < .001 |
| ENSSSCG00000015595 | *ATF3* | activating transcription factor 3 | 1.10 | 0.01 |
| ENSSSCG00000003155 | *PPP1R15A* | protein phosphatase 1, regulatory subunit 15A | 1.04 | 0.01 |
| ENSSSCG00000014362 | *HBEGF* | heparin-binding EGF-like growth factor | 0.99 | 0.02 |
| ENSSSCG00000005533 | *PTGS1* | uncharacterized protein | 0.95 | 0.03 |
| ENSSSCG00000009216 | *SPP1* | secreted phosphoprotein 1 | 0.91 | 0.02 |
