# Supplementary material for: Identification of Differentially Expressed Genes Associated with Litter Size in Berkshire Pig Placenta
Source: PLoS One. 2016 Apr 14;11(4):e0153311. doi: 10.1371/journal.pone.0153311 (PMC4831801; doi:10.1371/journal.pone.0153311)
Supplement: S3 Table — (DOCX) [file pone.0153311.s003.docx]

**S3 Table. List of down-regulated DEGs related to fecundity in LLG compared with SLG**

| **Accession** | **Symbol** | **Description** | **log_2_fc** | ***p*-value** |
| --- | --- | --- | --- | --- |
| ENSSSCG00000015326 | *COL1A2* | collagen, type I, alpha 2 | -1.00 | 0.01 |
| ENSSSCG00000008701 | *LREAP1* | low density lipoprotein receptor-related protein associated protein 1 | -1.02 | 0.01 |
| ENSSSCG00000009545 | *COL4A2* | collagen, type IV, alpha 2 | -1.04 | 0.01 |
| ENSSSCG00000016034 | *COL3A1* | collagen, type III, alpha 1 | -1.04 | 0.01 |
| ENSSSCG00000012083 | *RIPK4* | receptor-interacting serine-threonine kinase 4 | -1.05 | 0.01 |
| ENSSSCG00000016784 | *ANKH* | ANKH inorganic pyrophosphate transport regulator | -1.06 | 0.01 |
| ENSSSCG00000012832 | *MXRA5* | matrix-remodelling associated 5 | -1.08 | 0.01 |
| ENSSSCG00000022289 | *PCDH1* | protocadherin 1 | -1.09 | 0.01 |
| ENSSSCG00000003371 | *GPR153* | G protein-coupled receptor 153 | -1.12 | 0.01 |
| ENSSSCG00000005751 | *COL5A1* | collagen, type V, alpha 1 | -1.12 | 0.01 |
| ENSSSCG00000009513 | *SLC15A1* | solute carrier family 15 (oligopeptide transporter), member 1 | -1.18 | < .001 |
| ENSSSCG00000004012 | *THBS2* | thrombospondin 2 | -1.20 | 0.01 |
| ENSSSCG00000017192 | *EVPL* | envoplakin | -1.20 | < .001 |
| ENSSSCG00000022317 | *SLC38A10* | solute carrier family 38, member 10 | -1.21 | < .001 |
| ENSSSCG00000007927 | *PPL* | periplakin | -1.25 | < .001 |
| ENSSSCG00000011850 | *MUC4* | mucin 4, cell surface associated | -1.25 | < .001 |
| ENSSSCG00000010479 | *RBP4* | retinol binding protein 4, plasma | -1.26 | 0.01 |
| ENSSSCG00000008695 | *MFSD10* | major facilitator superfamily domain containing 10 | -1.26 | 0.01 |
| ENSSSCG00000012394 | *GJB1* | gap junction protein, beta 1, 32kDa | -1.26 | 0.01 |
| ENSSSCG00000005610 | *SLC2A8* | uncharacterized protein | -1.28 | 0.01 |
| ENSSSCG00000025423 | *KCNK5* | potassium channel, two pore domain subfamily K, member 5 | -1.28 | < .001 |
| ENSSSCG00000023404 | *FAM89A* | family with sequence similarity 89, member A | -1.32 | 0.01 |
| ENSSSCG00000021610 | *CHPF* | chondroitin polymerizing factor | -1.32 | < .001 |
| ENSSSCG00000017012 | *SLIT3* | slit guidance ligand 3 | -1.34 | < .001 |
| ENSSSCG00000028878 | *BCAR1* | breast cancer anti-estrogen resistance 1 | -1.34 | < .001 |
| ENSSSCG00000013079 | *DAGLA* | diacylglycerol lipase, alpha | -1.35 | 0.01 |
| ENSSSCG00000011355 | *COL7A1* | collagen, type VII, alpha 1 | -1.35 | 0.01 |
| ENSSSCG00000012699 | *FHL1C* | four and a half LIM domains 1 protein, isoform C | -1.37 | < .001 |
| ENSSSCG00000007485 | *BCAS1* | breast carcinoma amplified sequence 1 | -1.39 | < .001 |
| ENSSSCG00000014625 | *TRIM3* | tripartite motif containing 3 | -1.40 | 0.01 |
| ENSSSCG00000008550 | *SLC5A6* | solute carrier family 5 (sodium/multivitamin and iodide cotransporter), member 6 | -1.41 | 0.01 |
| ENSSSCG00000025924 | *IGFBP5* | uncharacterized protein | -1.42 | < .001 |
| ENSSSCG00000013614 | *CNN1* | calponin 1, basic, smooth muscle | -1.48 | < .001 |
| ENSSSCG00000013896 | *MPV17L2* | MPV17 mitochondrial membrane protein-like 2 | -1.50 | 0.01 |
| ENSSSCG00000008004 | *FAM173A* | family with sequence similarity 173, member A | -1.55 | 0.01 |
| ENSSSCG00000005930 | *SLC45A4* | solute carrier family 45, member 4 | -1.57 | < .001 |
| ENSSSCG00000005177 | *SH3GL2* | SH3-domain GRB2-like 2 | -1.60 | 0.01 |
| ENSSSCG00000009672 | *SCARA5* | scavenger receptor class A, member 5 | -1.60 | < .001 |
| ENSSSCG00000008557 | *EMILIN1* | elastin microfibril interfacer 1 | -1.65 | < .001 |
| ENSSSCG00000007530 | *PPP1R3D* | protein phosphatase 1 regulatory subunit 3 | -1.87 | < .001 |
| ENSSSCG00000007574 | *SDK1* | sidekick cell adhesion molecule 1 | -1.90 | < .001 |
| ENSSSCG00000014232 | *LOX* | lysyl oxidase | -1.90 | < .001 |
| ENSSSCG00000007816 | *IL21R* | interleukin 21 receptor | -1.91 | < .001 |
| ENSSSCG00000025777 | *ER* | estrogen receptor | -1.93 | 0.01 |
| ENSSSCG00000002368 | *LTBP2* | latent transforming growth factor beta binding protein 2 | -1.94 | < .001 |
| ENSSSCG00000008294 | *ACTG2* | actin, gamma 2, smooth muscle, enteric | -2.04 | < .001 |
| ENSSSCG00000024018 | *SLC16A3* | solute carrier family 16 (monocarboxylate transporter), member 3 | -2.06 | < .001 |
| ENSSSCG00000029613 | *SYNM* | synemin, intermediate filament protein | -2.07 | < .001 |
| ENSSSCG00000004632 | *GLDN* | gliomedin | -2.08 | < .001 |
| ENSSSCG00000010437 | *PAPSS2* | 3'-phosphoadenosine 5'-phosphosulfate synthase 2 | -2.11 | < .001 |
| ENSSSCG00000023215 | *MAOB* | monoamine oxidase B, nuclear gene encoding mitochondrial protein | -2.12 | < .001 |
| ENSSSCG00000024914 | *BF* | complement factor B | -2.18 | 0.01 |
| ENSSSCG00000002444 | *FBLN5* | fibulin 5 | -2.19 | < .001 |
| ENSSSCG00000017445 | *KRT13* | keratin 13, type I | -2.26 | < .001 |
| ENSSSCG00000002265 | *FAM174B* | family with sequence similarity 174, member B | -2.27 | < .001 |
| ENSSSCG00000023662 | *CHST3* | carbohydrate (chondroitin 6) sulfotransferase 3 | -2.31 | < .001 |
| ENSSSCG00000002696 | *VAT1L* | vesicle amine transport 1-like | -2.35 | 0.01 |
| ENSSSCG00000002527 | *ANKRD9* | ankyrin repeat domain 9 | -2.40 | < .001 |
| ENSSSCG00000002545 | *ZFYVE21* | zinc finger, FYVE domain containing 21 | -2.41 | 0.01 |
| ENSSSCG00000025578 | *ALDH1A2* | aldehyde dehydrogenase 1 family, member A2 | -2.43 | < .001 |
| ENSSSCG00000000892 | *HAL* | Histidine ammonia-lyase | -2.45 | < .001 |
| ENSSSCG00000026547 | *SLC45A3* | solute carrier family 45, member 3 | -2.49 | < .001 |
| ENSSSCG00000007549 | *CYP2W1* | cytochrome P450, family 2, subfamily W, polypeptide 1 | -2.59 | < .001 |
| ENSSSCG00000000664 | *A2ML1* | alpha-2-macroglobulin-like 1 | -2.61 | < .001 |
| ENSSSCG00000009250 | *PRKG2* | cGMP-dependent protein kinase | -2.66 | < .001 |
| ENSSSCG00000010947 | *FBP2* | fructose-1,6-bisphosphatase 2 | -2.73 | < .001 |
| ENSSSCG00000015249 | *ADAMTS8* | ADAM metallopeptidase with thrombospondin type 1 motif, 8 | -2.80 | < .001 |
| ENSSSCG00000029675 | *MMP8* | matrix metallopeptidase 8 | -3.05 | < .001 |
| ENSSSCG00000006604 | *CRNN* | cornulin | -3.10 | < .001 |
| ENSSSCG00000014633 | *PRKCDBP* | protein kinase C, delta binding protein | -3.14 | < .001 |
| ENSSSCG00000016728 | *IGFBP1* | insulin-like growth factor binding protein 1 | -3.16 | < .001 |
| ENSSSCG00000013501 | *CREB3L3* | cAMP responsive element binding protein 3-like 3 | -3.17 | < .001 |
| ENSSSCG00000009722 | *SPOCK3* | sparc/osteonectin, cwcv and kazal-like domains proteoglycan (testican) 3 | -3.20 | < .001 |
| ENSSSCG00000002901 | *UPK1A* | uroplakin 1A | -3.22 | < .001 |
| ENSSSCG00000012852 | *CDHR5* | cadherin-related family member 5 | -3.44 | < .001 |
| ENSSSCG00000007507 | *PCK1* | phosphoenolpyruvate carboxykinase 1 (soluble) | -3.47 | < .001 |
| ENSSSCG00000005845 | *TOR4A* | torsin family 4, member A | -3.50 | < .001 |
| ENSSSCG00000023949 | *PIM3* | pim-3 oncogene | -3.50 | < .001 |
| ENSSSCG00000005832 | *MAMDC4* | MAM domain containing 4 | -3.58 | < .001 |
| ENSSSCG00000010036 | *SLC5A1* | solute carrier family 5 (sodium/glucose cotransporter), member 1 | -3.78 | < .001 |
| ENSSSCG00000017433 | *KRT14* | keratin 14, type I | -3.83 | < .001 |
| ENSSSCG00000012576 | *CHRDL1* | chordin-like 1 | -4.16 | 0.01 |
| ENSSSCG00000016196 | *VIL1* | villin-1 | -4.18 | < .001 |
| ENSSSCG00000020694 | *DSG1* | desmoglein 1 | -4.36 | < .001 |
| ENSSSCG00000013377 | *USH1C* | usher syndrome 1C | SLG only | 0.01 |
| ENSSSCG00000011453 | *ITIH4* | inter-alpha-trypsin inhibitor heavy chain H4 | -4.99 | < .001 |
| ENSSSCG00000005843 | *LCN15* | lipocalin 15 | SLG only | 0.01 |
| ENSSSCG00000024610 | *KRT4* | keratin 4, type II | -5.03 | < .001 |
| ENSSSCG00000014055 | *CDHR2* | cadherin-related family member 2 | -5.07 | < .001 |
| ENSSSCG00000009558 | *F10* | coagulation factor X protein (LOC733662) | -5.16 | < .001 |
| ENSSSCG00000017459 | *KRT20* | keratin, type I cytoskeletal 20 | -5.23 | 0.01 |
| ENSSSCG00000006106 | *CDH17* | cadherin 17, LI cadherin (liver-intestine) | -5.24 | < .001 |
| ENSSSCG00000026978 | *ROS1* | ROS proto-oncogene 1 , receptor tyrosine kinase | -5.54 | < .001 |
| ENSSSCG00000010792 | *PRAP1* | proline-rich acidic protein 1 | -5.54 | < .001 |
| ENSSSCG00000009942 | *DAO* | D-amino acid oxidase | SLG only | < .001 |
| ENSSSCG00000011862 | *MUC13A* | mucin 13, cell surface associated | SLG only | < .001 |
| ENSSSCG00000006712 | *REG4* | regenerating islet-derived family, member 4 | SLG only | < .001 |
| ENSSSCG00000016746 | *NPC1L1* | NPC1-like 1 | -6.09 | < .001 |
| ENSSSCG00000000248 | *KRT5* | keratin 5, type II | -6.19 | < .001 |
| ENSSSCG00000006820 | *EPS8L3* | EPS8-like 3 | SLG only | < .001 |
| ENSSSCG00000017444 | *KRT15* | keratin 15, type I | -6.20 | < .001 |
| ENSSSCG00000024092 | *KRT77* | keratin 77, type II | SLG only | < .001 |
| ENSSSCG00000017120 | *SLC6A19* | transporter | SLG only | < .001 |
| ENSSSCG00000008595 | *APOB* | uncharacterized protein | SLG only | < .001 |
| ENSSSCG00000007371 | *HNF4A* | hepatocyte nuclear factor 4, alpha | SLG only | < .001 |
| ENSSSCG00000021728 | *LGALS2* | lectin, galactoside-binding, soluble, 3 | SLG only | < .001 |
| ENSSSCG00000003080 | *IGSF23* | immunoglobulin superfamily, member 23 | SLG only | < .001 |
| ENSSSCG00000011663 | *RBP2* | retinol binding protein 2, cellular | SLG only | < .001 |
| ENSSSCG00000016247 | *TM4SF20* | transmembrane 4 L six family member 20 | SLG only | < .001 |
| ENSSSCG00000000251 | *KRT1* | keratin 1, type II | -10.20 | < .001 |
